# Severe COVID-19: what have we learned with the immunopathogenesis?

**DOI:** 10.1186/s42358-020-00151-7

**Published:** 2020-09-22

**Authors:** Bruno Bordallo, Mozart Bellas, Arthur Fernandes Cortez, Matheus Vieira, Marcelo Pinheiro

**Affiliations:** 1 Departament of Internal Medicine / Emergence, Hospital Universitário Antônio Pedro / Univesidade Federal Fluminense, Niterói, RJ Brazil; 2 Departament of Internal Medicine / Emergence, Hospital Universitário Antônio Pedro / Universidade Federal Fluminense, Niterói, RJ Brazil; 3 Hospital Universitário Gaffré e Guinle / Universidade Federal do Estado do Rio de Janeiro, Internal Medicine Departament, Rio de Janeiro, RJ Brazil; 4 Departament of Internal Medicine, Hospital Universitário Clementino Fraga Filho, Rio de Janeiro, RJ Brazil; 5 Departament of Rheumatology, Universidade Federal de São Paulo, Sao Paulo, SP Brazil

**Keywords:** COVID-19, SARS-CoV-2, Immunology, Inflammation, Cytokine storm, Cytokine, Macrophage activation syndrome, Thrombosis

## Abstract

The COVID-19 outbreak caused by severe acute respiratory syndrome coronavirus 2 (SARS-CoV-2) has become a global major concern. In this review, we addressed a theoretical model on immunopathogenesis associated with severe COVID-19, based on the current literature of SARS-CoV-2 and other epidemic pathogenic coronaviruses, such as SARS and MERS. Several studies have suggested that immune dysregulation and hyperinflammatory response induced by SARS-CoV-2 are more involved in disease severity than the virus itself.

Immune dysregulation due to COVID-19 is characterized by delayed and impaired interferon response, lymphocyte exhaustion and cytokine storm that ultimately lead to diffuse lung tissue damage and posterior thrombotic phenomena.

Considering there is a lack of clinical evidence provided by randomized clinical trials, the knowledge about SARS-CoV-2 disease pathogenesis and immune response is a cornerstone to develop rationale-based clinical therapeutic strategies. In this narrative review, the authors aimed to describe the immunopathogenesis of severe forms of COVID-19.

## Background

The severe acute respiratory syndrome coronavirus 2 (SARS-CoV-2), a positive-sense single-stranded RNA-enveloped virus, is the causative agent of coronavirus disease 2019 (COVID-19), being first identified in Wuhan, China, in December 2019. Previously, other epidemic coronavirus such as severe acute respiratory syndrome coronavirus (SARS-CoV) in 2002 and the middle-east respiratory syndrome coronavirus (MERS-CoV) in 2012, had serious impact on human health and warned the world about the possible reemergence of new pathogenic strains [1]. Despite being a new virus, several common morpho-functional characteristics have been reported between SARS-CoV and the SARS-CoV-2, including the interaction of the viral spike (S) glycoprotein with the human angiotensin converting enzyme 2 (ACE2). These similarities may help understanding some pathophysiological mechanisms and pointing out possible therapeutic targets.

The first step for SARS-CoV-2 entry into the host cell is the interaction between the S glycoprotein and ACE2 on cell surface. Since the latter acts as a viral receptor, the virus will only infect ACE2 expressing cells, notably type II pneumocytes. These cells represent 83% of the ACE2-expressing cells in humans, but cells from other tissues and organs, such as heart, kidney, intestine and endothelium, can also express this receptor [2]. A host type 2 transmembrane serine protease, TMPRSS2, facilitates virus entry by priming S glycoprotein. TMPRSS2 entails S protein in subunits S1/S2 and S2´, allowing viral and cellular membrane fusion driven by S2 subunit [3]. Once inside the cell viral positive sense single strand RNA is translated into polyproteins that will form the replicase-transcriptase complex. This complex function as a viral factory producing new viral RNA and viral proteins for viral function and assembly [4]. Considering these particularities, the infection first begins on upper respiratory tract mucosa and then reaches the lungs. The primary tissue damage is related to the direct viral cytopathic effects. At this stage, the virus has the potential to evade the immune system, where an inadequate innate immune response can occur, depending on the viral load and other unknown genetic factors. Subsequently, tissue damage is induced by additional mechanisms derived from a dysregulated adaptive immune response [5].

Although most of COVID-19 cases have a mild clinical course, up to 14% can evolve to a severe form, with respiratory rate ≥ 30/min, hypoxemia with pulse oxygen saturation ≤ 93%, partial pressure of arterial oxygen to fraction of inspired oxygen ratio < 300 and/or pulmonary infiltrates involving more than 50% of lung parenchyma within 24 to 48 h. Up to 5% of the cases can be critical, evolving with respiratory failure, septic shock and/or multiple organ dysfunction, presumably driven by a cytokine storm [6]. Host characteristics, including aging (immunosenescence) and comorbidities (hypertension, diabetes mellitus, lung and heart diseases) may influence the course of the disease [7]. The false paradox between inflammation and immunodeficiency is highlighted by the severe form of COVID-19. Thus, severe pneumonia caused by SARS-CoV-2 is marked by immune system dysfunction and hyperinflammation leading to acute respiratory distress syndrome (ARDS), macrophage activation, hypercytokinemia and coagulopathy [8].

Herein, we aim to review the factors related to the dysregulated immune response against the SARS-CoV-2, along with its relation with severe forms of COVID-19, namely ARDS and cytokine storm (CS).

## Virus and host interaction

### Mechanism of invasion and cell damage of SARS-CoV-2

The virus penetrates the body through the inhalation of contaminating particles, mainly droplets and aerosols from infected hosts, first lodging in the upper respiratory tract and then reaching the lungs. SARS-CoV-2 uses ACE2 to infect epithelial cells form pharynx, larynx, alveolus (type II pneumocytes), alveolar macrophages and endothelial cells [2, 9].

Glycoprotein S is present in homotrimers on the viral surface. It is divided into two subunits, S1 that bind ACE2, and S2 that fuses with the cell membrane. The S1 and S2 subunits sites are cleaved by a transmembrane serine protease called TMPRSS2. SARS-CoV-2 can also use endosomal proteases cathepsin B and L for S protein priming in TMPRSS2 non expressing cells [3]. After glycoprotein S-ACE2 interaction and membrane fusion, the virus enters the cell using the endossomic compartment alongside with the ACE2 receptor. This early endosome becomes a late endosome and merges with a lysosome to form an endolysosome. At this moment, the virus leaves the endolysosome and reaches the cytoplasm, where its viral genome will be translated [3].

Hydroxychloroquine and chloroquine are widely used to treat patients with rheumatic diseases, especially systemic erythematosus lupus, with a known antiviral effect against SARS and SARS-CoV-2 in vitro. In addition to anti-inflammatory effects by disrupting endosomal toll like receptors signaling, the endolysosomal pH increase would also hamper the fusion of virus membrane with the endosomal membrane inhibiting the viral entry into the cell [10]. Unfortunately, the antiviral action of hydroxychloroquine did not occur in vivo. Clinical studies have shown that there is no benefit of these antimalarial drugs for treating hospitalized patients or in post-exposure prophylaxis [11, 12].

Regarding pathological findings, typical characteristics of ARDS such as epithelial desquamation, hyaline membrane and pulmonary edema are seen, but cytopathic induced damage is also noted. Despite the fact that no intracytoplasmic viral inclusion was seen, multinucleated syncytial cells with atypical enlarged pneumocytes were observed, characterized by large nuclei, amphophilic granular cytoplasm, and prominent nucleoli, which indicates direct viral damage. Another important feature seen was the pulmonary infiltration of mononuclear cells and neutrophils [13]. Intense replication of SARS-CoV-2 leading to inflammatory cell death is an essential component of COVID-19 pathogenesis mainly in the initial phases but can be present along all disease process [14].

The fast intracellular viral replication leads to apoptosis and pyroptosis of infected cells, causing capillary leakage and the release of several pro-inflammatory cytokines. Pyroptosis is a pro-inflammatory cell death process induced by the assembly of a multiprotein complex called inflammasome [15]. The inflammasome activation is a well-known mechanism of tissue damage related to viral infection. It can be triggered by endoplasmic reticulum stress response or by ion influx through viroporins [16, 17].

Viroporin are viral proteins that undergo oligomerization forming ions channel and have been related to many functions in multiple stages of viral life cycle and enhancement of pathogenic effects. Coronaviruses encodes two viroporins: E and 3a. SARS-CoV-2 viroporin E acts as a Ca^2+^ selective ion channel that also activates the nucleotide-binding domain, leucine-rich repeat and pyrin domain-containing protein 3 (NLRP3) inflammasome [17–19]. Viroporin 3a forms a homotetramer complex that work as an ion channel to promote virus release. The viroporin 3a ion channel leads to K^+^ efflux and mitochondrial reactive oxygen species production that activate NLRP3 inflammasome. Thus, both viroporins are responsible for inflammasome activation, with subsequent release of IL-1ß and pyroptosis [18].

Inflammasome promotes the proteolytic cleavage of pro-IL-1β into the active form IL-1β, a pro-inflammatory cytokine, as well as cleavage of Gasdermin-D into Gasdermin N, forming pores and inducing the inflammatory cell death [20]. The death of the infected cell releases cellular and viral fragments known as damage associated molecular patterns (DAMPs) and pathogens associates molecular patterns (PAMPs), respectively, that may be sensed by Toll-like-receptor (TLR) of myeloid cells, and leads to the production and release of more inflammatory cytokines [21]. In addition, IL-1β triggers other pro-inflammatory cytokines through a paracrine way [22]. The probable role of pyroptosis and high levels of IL1- β in the pathogenesis of severe COVID-19 provided the rational to development of clinical trials to address the efficacy and safety of anti-IL-1 targeted therapy [23]. In a prospective non-randomized trial compared with a historical control cohort, anakinra, a recombinant, nonglycosylated human interleukin-1 receptor inhibitor, was associated with lower need for invasive mechanical ventilation and mortality rate in patients with severe forms of COVID-19 [24].

Despite enthusiasm for cytokine targeted drugs, the use of more available and safer drugs has been pursued. In this context, colchicine is a low-cost, widely available drug to treat diseases with an auto-inflammatory phenotype such as familial Mediterranean fever, Behçet’s disease and gouty arthritis. Anti-inflammatory properties of colchicine, mainly neutrophil chemotaxis inhibition and blockage of NLRP3 inflammasome are important targets in COVID-19 pathogenesis and are being evaluated in clinical trials [25].

### ACE2 downregulation

**Fig. 1:**
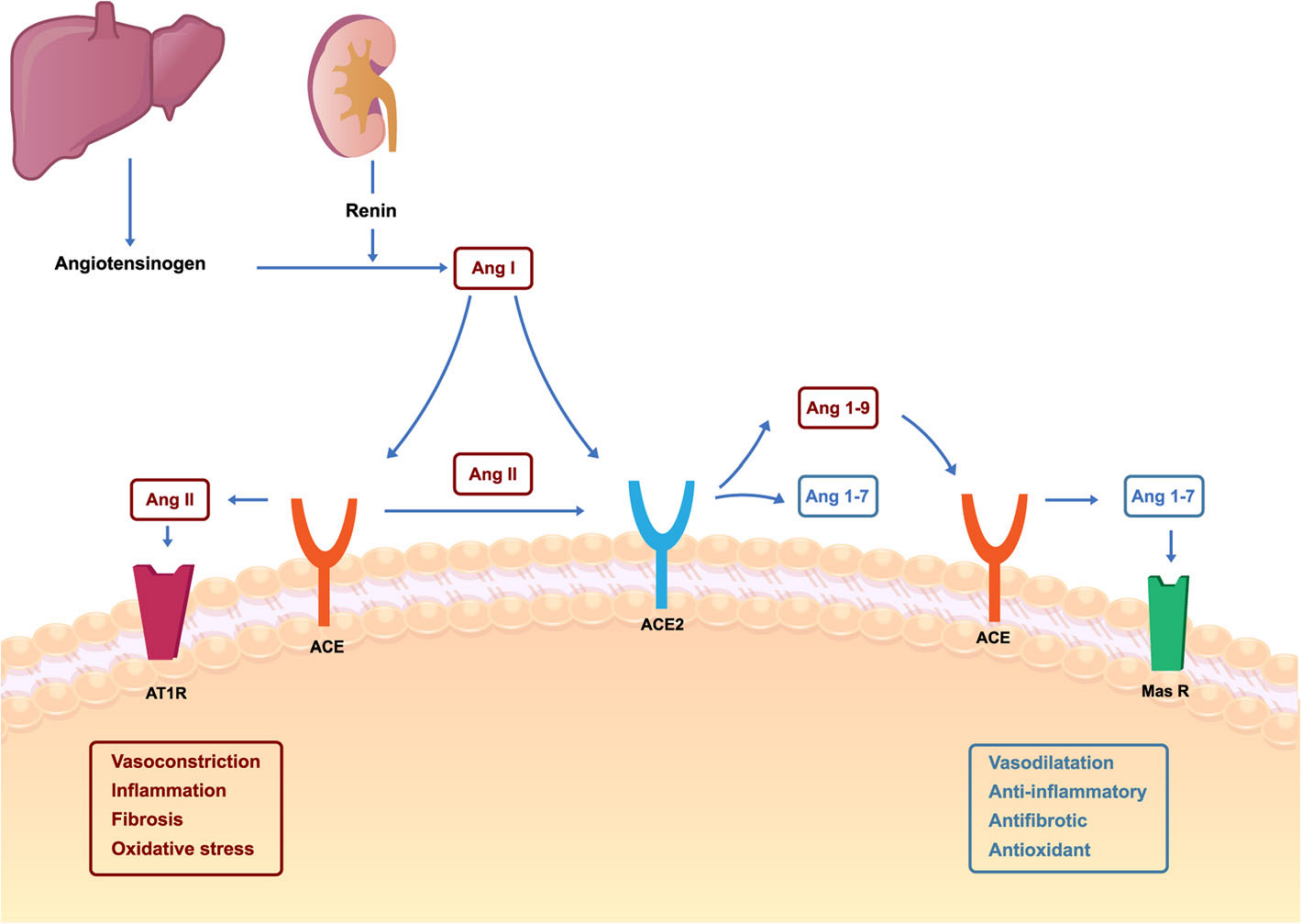
Renin Aldosterone Angiotensin System in COVID-19. Angiotensinogen is produced by the liver and converted to angiotensin I by renin (produced by juxtaglomerular renal cells) in response to hypovolemia. Angiotensin I can be either converted by ACE into Angiotensin II or metabolized to Angiotensin 1–9 by ACE2. Moreover, angiotensin I can also be converted to Angiotensin 1–7 by ACE2. The Ang 1–7/MAS axis can have beneficial effects on lungs, while the Ang-II / AT1a axis have deleterious effects. Adapted from Yan, T, Xiao, R, Lin, G. 2020 [28]

Another important mechanism of tissue damage is mediated by downregulation of ACE2 and its protective functions. ACE2 is an enzyme that cleaves angiotensin II (Ang II) and angiotensin I (Ang I) into angiotensin 1–7 and angiotensin 1–9, respectively [26]. It can be found as a transmembrane protein, acting as a receptor to SARS-CoV-2 attachment, or as a soluble protein. Angiotensin II has several deleterious effects that can contribute to lung injury through the AT1a receptor, comprising vasoconstriction, cell proliferation, inflammation, increased vascular permeability and fibrosis. On the other hand, Ang 1–7 present beneficial effects through signaling by Mas receptor, that leads to vasodilatation, anti-inflammatory and anti-fibrosis activity [27]. ACE2 receptor-mediated virus endocytosis leads to its downregulation, which might contribute to vasoconstriction, inflammation, vascular leakage and lung injury [27] (Fig. 1). In a study with ARDS murine model, the administration of recombinant ACE2 mitigated the progression to severe acute lung injury [29]. Similarly, another study with ACE2 knockout mice demonstrated more severe lung inflammation after acid inhalation when compared to wild-type mice [30].

A hypothesis that may partially explain the propensity of some individuals to develop more or less likely the severe forms of COVID-19 is concerning the ACE2 expression. Blood ACE2 activity is inversely proportional to body mass index and blood pressure [31], while estrogens act in its upregulation [32]. Also, children have higher levels of circulating ACE2. These findings may justify why women and children are less prone than adult or elderly men to develop the severe forms of the disease, especially those with obesity and hypertension [33]. However, other mechanisms explain its pathogenicity. The immune response plays an important role.

### Immune and inflammatory response

Innate immunity cells such as macrophages, monocytes and dendritic cells are activated after recognizing DAMPs or PAMPs through their pattern recognition receptor (PRRs). This interaction trigger intracellular pathways that activates transcription factors and leads to cytokine, chemokine and adhesion molecule expression [21].

Viral PAMPs, specially viral RNA are sensed by immune system by endosomal TLRs, mainly TLR-7, and cytosolic nucleic acid sensors like retinoic-acid inducible gene 1 (RIG-1) and melanoma differentiation-associated protein 5 (MDA5), and that latter mechanism seems to be impaired in elderly [34]. SARS-CoV-2 S-glycoprotein can be sensed by TLR-4, activating the nuclear factor kappa-B (NF-kB) pathway [35]. The activation of TLRs and RIG-1 like receptors (RLR’s) leads to the production and release of pro-inflammatory cytokines and interferons through the activation of the transcription factors NF-kB and Interferon Regulatory Factor (IRF) 3 and 7 [35].

In parallel, mainly dendritic cells present antigens to T helper cells and B lymphocytes in the lymphoid follicles through major histocompatibility complex (MHC) class II, triggering a more specific cellular and humoral response with effector cytotoxic lymphocytes differentiation and antibodies production, respectively [36, 37].

Alongside with mechanisms of activation of immune system, several mechanisms that suppress inflammatory process takes place in order to limit host damage by the immune system. Among them, NK cells may play an interesting role in immunomodulating the response against COVID-19. Activated myeloid cells upregulate the NKG2D ligands expression, triggering NK cells to kill them and hampering the resolution of inflammation by a contra-regulatory immune mechanism [38].

When there is an effective immune response, optimized viral clearance limits disease progression. In addition, viral antigens presentation by innate immunity cells to lymphocytes in the lymphoid organs enhances adaptive immunity and stimulates humoral immunity. B cells differentiate into plasma cells and produce antibodies that are able to neutralize and contain viral spreading. The coordinated and efficient immune response limits the infection.

## Risk factors for development of severe forms

### Age and sex

Advanced age hampers an efficient immune response. Several mechanisms are proposed to favor greater susceptibility to infections, poor vaccine response and degenerative inflammatory diseases. Immunosenescence evolves with reduced naïve T cells pool and memory T cells accumulation [39]. In the elderly, late differentiated effector memory T cells present a senescent phenotype with poor reproductive capacity, but higher capacity of pro-inflammatory cytokines production [40]. This concept is called inflammaging [41] and it is modulated by different factors, such as hormonal, microbiome, nutritional and comorbidities. Aged primates showed enhancement of NF-kB pathway activation, presenting a pro-inflammatory cytokine profile [42]. On the other hand, elderly is known to present an inefficient antiviral response due to an impaired cytokine release by DC. These cells are the most important source of type I interferons, which plays a major role in early antiviral response [39].

Interestingly, a higher male lethality is seen in COVID-19. Hormonal factors could be implicated, since chemotactic factors involved in neutrophils and monocytes recruitment such as CXCL1 and CCL20 are regulated by an androgen receptor. Noteworthy, estrogen receptor regulates immune response by enhancing interferon production and antiviral response. The selective estrogen receptor modulator (toremifene) have been proposed as a potential drug to treat coronavirus infection [43, 44].

### Comorbidities

The majority of patients that develop severe forms of COVID-19 with CS, mononuclear lung cell infiltration and prothrombotic state have two or more comorbidities [45]. Despite being well described risk factors, the exact mechanism by which comorbidities (e.g. hypertension, diabetes and obesity) affect the clinical course is unclear.

An American study with 5700 patients revealed that the median age of the hospitalized patients was 63 years, 57% of them had hypertension, 42% were obese and 34% had diabetes [45]. Metabolic syndrome and obesity evolve with chronic inflammation due to higher NF-κB activity and pro-inflammatory cytokine production, such as IL-1, interleukin-6 (IL-6) and tumor necrosis factor-α (TNF- α) [46]. Moreover, perturbation of hormonal and metabolic homeostasis seen in obesity could impair antiviral response, and bronchial epithelial cells from obese patients showed reduction of the interferon responses, increasing viral replication [47].

Patients with hypertension present with endothelial cells dysfunction and immunometabolic modifications that contribute to higher baseline inflammatory cytokine serum levels [48]. Diabetes is associated with higher risk of infection, and also present a pro-inflammatory cytokine profile, being considered as a risk factor for mortality in several viral pneumonias such as influenza (H1N1), SARS-CoV and MERS-CoV [49, 50]. Further studies are necessary to better understand the pathophysiological mechanisms through which comorbidities impact on severe COVID-19.

### Laboratory abnormalities

Several laboratory risk factors for the development of severe forms have been identified. Most of them are related to hyperinflammation, immune dysregulation and hypercoagulability/ hyperfibrinolysis [51]. A Chinese retrospective, multicenter cohort study, with 191 patients showed higher mortality risk in patients with diabetes, coronary heart disease, advanced age, lymphopenia, leukocytosis, and elevated alanine aminotransferase, lactate dehydrogenase, high-sensitivity cardiac troponin I, creatine kinase, d-dimer, serum ferritin, IL-6 [52].

High ferritin serum levels are a well-known inflammation biomarker. Hyperferritinemic syndromes encompasses heterogeneous life-threatening conditions characterized by hyperinflammation and high ferritin levels [53]. Ferritin is not only a biomarker of acute phase response, but also seems to play a pathogenic role in inflammation. In patients with COVID-19, it is associated to poor prognosis, severe disease and mortality [52, 54].

IL-6 is an important inflammatory biomarker and is correlated with acute phase reactants. Several studies showed that high IL-6 plasmatic levels were related to COVID-19 severity [55], suggesting that IL-6 inhibitors, like Tocilizumab, could be a potential therapy for severe COVID-19 patients [23]. The pathogenic aspects regarding the IL-6 in COVID-19 will be addressed below.

D-dimer is a fibrin degradation product and its rising levels has been related to activation of coagulation and subsequent fibrinolysis. In addition, in a retrospective cohort, D-dimer plasmatic levels above 2.0 μg/mL, on admission, were considered as a relevant mortality predictor. More recently, a systematic review on biomarkers related to COVID-19 severity showed D-dimer has strong association with mortality [56]. Therefore, anticoagulation therapeutic strategies have been justified by higher thrombotic phenomena incidence [57].

Lymphopenia, which is seen in up to 72–85% of severe cases is a hallmark of COVID-19. Severe cases tend to have lower lymphocytes counts, higher leukocytes count and high neutrophil-lymphocyte-ratio (NLR). All lymphocytes subsets, including CD4+ T cells, CD8+ T cells, B cells and natural killer cells decreased in COVID-19, especially in patients who develop severe forms. CD8+ T cells and CD4+/CD8+ ratio showed a significant association with the inflammatory status in COVID-19 and were independent predictors for poor outcomes [58]. Patients with COVID-19 have lower level of regulatory T cells, mostly in severe cases [59].

Lymphopenia mechanism is not completely understood. One of the proposed mechanism is lung infiltration with lymphocyte sequestration [60]. Indeed, it is well-documented that genes related do lymphocytes apoptosis such as annexin V and exhaustion-related genes are upregulated [58, 61, 62]. Lymphocyte exhaustion will be discussed below.

## Immunopathogenesis of severe forms

### Inadequate interferon response

It is well-established that interferons, mainly types I and III, are crucial in promoting antiviral state, providing an effective viral clearance. Multiple pathways are involved in this complex response, including upregulation of Interferon Stimulated Genes (ISGs) that enhances MHC-I presentation, as well as immune cells activation, apoptosis of infected cells and production of antiviral proteins that suppress viral replication [35].

Once type I interferon binds to its receptor (IFNAR1 and INFAR2), it activates the JAK/ STAT signaling pathway, through which non-receptors tyrosine kinase JAK1 and TYK2 will determine the phosphorylation of STAT1 and STAT2 and finally the expression of ISGs [63]. Type III interferon (also known as lambda interferon) shares similar effects and intracellular signaling pathways with type I interferon, although using a different membrane receptor present only in mucosal sites [64]. Thus, interferons I and III are essential in the early antiviral response. Type II interferon (i.e. gamma interferon), in turn, plays a fundamental role on intracellular microorganism defense, inducing macrophage activation and Th1 response [65]. Dead and apoptotic cells can be phagocyted by macrophages (M0) and dendritic cells (DC) leading to antigen presentation by MHC-II to TCD4+ cells enhancing the adaptive immune response [66]. Moreover, viral antigens can be expressed by MHC-I molecules of infected cells activating cytotoxic TCD8+ cells and cellular immune response [67].

A striking feature of coronaviruses is their ability to inhibit interferon action. Although the SARS-CoV-2 has 3.2-fold higher replication rate, it induced lesser production and release of types I, II and III interferons when compared with SARS-CoV infection. Therefore, SARS-CoV-2 can reach high viral loads before activating an innate immune response [68].

Several coronavirus non-structural proteins (NsP), like Nsp1, Nsp3, Nsp14, ORF3b, ORF6 and the structural M and N proteins could impair the interferon production, signalization and antiviral activity through many different mechanisms [35]. Among some trickeries used by coronaviruses to evade the immune system is the fact that they replicate within double membrane vesicles, hiding the viral RNA from cytosolic RIG-like receptors, a characteristic of the *Nidovirus* order [69].

In a SARS-COV murine model, Channappanavar et al. demonstrated that the Interferon (IFN) late response is associated with severe forms of pneumonia and ARDS. It is characterized by accumulation of pathogenic inflammatory monocyte-macrophages (IMMs) that results in high cytokine/chemokine concentration in lung tissue, vascular leakage, and impaired virus-specific T cell responses [70].

The same authors reported the relevance of interferon kinetics in MERS murine infection. When administered on the first day after infection, IFN-beta had a protective effect due to increased viral clearance. However, if administered later, not only was the viral clearance not induced, but also an increased pulmonary inflammatory infiltration by macrophages and monocytes happened, resulting in a CS [71].

Thus, an inadequate antiviral response, either by viral immune evasive mechanisms or by an impaired host response to interferon, leads to an insufficient viral clearance, with higher risk of developing pneumonia, ARDS and CS [62]. This immune dysregulation phenotype found in COVID-19 infection is characterized by impaired interferon I response and downregulation of interferon stimulated genes.

**Fig. 2:**
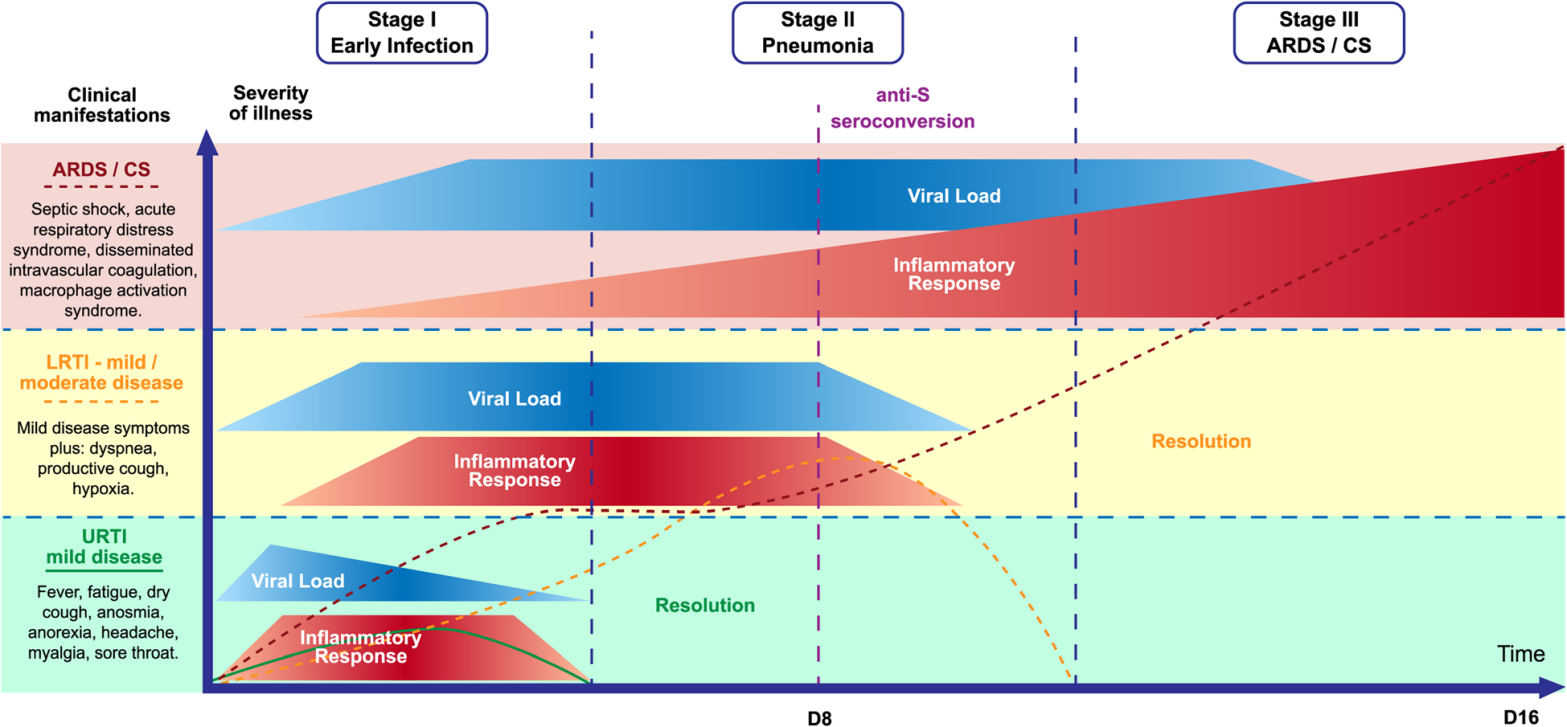
Spectrum of severity and stages of COVID-19. UPRT – Upper Respiratory Tract Infection; LRTI – Lower Respiratory Tract Infection; ARDS – Acute Respiratory Distress Syndrome; CS – Cytokine Storm

An impaired antiviral interferon response may lead to a high viral load at the time of antibody formation. In SARS outbreak, seroconversion occurs on the eighth day of symptoms and it coincides with the worsening of the disease in 80% of cases. This dynamic process suggests that antibody-immune enhancement may plays an important role in the pathophysiology of COVID-19 severe forms [72] (Fig. 2).

### Humoral response and antibody-dependent enhancement

Humoral response is a pivotal component against viral infections. SARS-CoV-2 cause a strong B cell activation, maturation and antibody production. Neutralizing antibodies (Nab) against the receptor biding domain of glycoprotein S is present in the vast majority of patients with COVID-19 following infection. Nab block the viral-receptor interaction and inhibit the viral entrance in the host cell [73].

Antibody-dependent enhancement (ADE) is a phenomenon in which subneutralizing antibodies enhance the entry of virus into monocytes/macrophages and granulocytic cells through interaction with Fc and/or complement receptors [74]. Clinical deterioration associated with ADE is a well described phenomenon in several viral infections such as the Dengue, Zika, Ebola, Influenza and veterinary coronavirus that causes feline infectious peritonitis [75–79]. A prime example is the development of the most severe form of dengue, the dengue hemorrhagic fever (DHF), that develop in patients with previous infection by a different serotype. In this situation, the risk of DHF is enhanced due to the presence of subneutralizing antibodies against the previous serotype [75].

The fact that seasonal human coronaviruses such as NL63, 229E, OC43, HKU-1 are responsible for 8–18.4% of all respiratory tract infections, usually common cold help to explain the rationale behind the hypothesis of ADE in SARS-CoV-2 infection [80].

Viral entry via FC receptor can lead to productive infection, when virus can replicate inside myeloid cells or to unproductive infection when virus is destructed and no infective virus are released. Despite the fact that there is no evidence of SARS-CoV-2 replication inside myeloid cells, viral entry by Fc receptor, mainly Fc-gamma-RII (CD32) may lead to activation of endosomal TLR and release of proinflammatory cytokines. This phenomenon is known as antibody-immune-enhancement [81]. In the case of SARS-CoV-2, it is possible that a suboptimal humoral response leading to low titters of IgG anti-S or production of non-neutralizing antibodies can mediate antibody-immune enhancement [82].

Tseng et al. demonstrated that exposure to infectious SARS-CoV causes maturation and phenotypic alterations of dendritic cells enhancing T-cell-stimulatory capacity and cytokine release. In the same study, the authors showed that exposure to SARS-CoV led to diminished phagocytic capacity in macrophage and primes it leading to massive production and release of cytokines in response to low dose of LPS [81].

Noteworthy, the development of severe forms of disease occurs at the time of seroconversion in 80% of patients with SARS-CoV pneumonia [72]. In line with that, animal models actively immunized with anti-S IgG evidenced that these animals develop a more pronounced lung damage than non-immunized ones. This damage was mediated by lung infiltration by inflammatory monocytes and macrophages (IMM) [83].

### Lymphocyte exhaustion

It is well known that persistent infections by several viruses can lead to immune exhaustion. Besides lymphopenia, lymphocytes in COVID-19 severe forms also exhibit an exhausted phenotype, characterized by impaired effector functions [84]. These exhausted T cells are more frequently found in those with severe forms [85].

The CD8+ T cells role in the immune response to coronavirus was highlighted in a bronchoalveolar lavage fluid analysis from patients with COVID-19. In mild symptomatic cases, a highly expanded clonal CD8+ T cell population was found, suggesting that a robust adaptive cellular immune response was related to a better disease control [5]. CD8+ T and NK cells from COVID-19 patients have increased expression of the inhibitory receptor NKG2A compared to healthy controls. This altered expression is normalized in convalescent patients [84].

Genome-wide transcriptional signature from exhausted CD8+ T cells showed altered expression of inhibitory and co-stimulatory receptors such as PD-1 and LAG-3 [86]. In this light, immune checkpoint inhibitors could restore effector functions and improve viral clearance [87, 88].

T cell exhaustion is a cornerstone of COVID-19 cytokine storm, since T cell activity is crucial for virus clearance and innate immune inflammation shutdown [84]. The inability to eliminate the virus due to lymphocyte exhaustion is both the cause and consequence of a high antigenic stimulus. This scenario favors continuous myeloid cell stimulation and hyperinflammation.

### Inflammatory monocytes and macrophage lung infiltration

A minimally-invasive autopsy study demonstrated that inflammatory monocytes and macrophages accumulate in the lungs and are the likely source of pro-inflammatory cytokines and chemokines. Part of them were positive for SARS-CoV-2 by immunohistochemical staining [89].

The presence of anti-S antibody prior to the viral clearance appears to play a role in severe acute lung injury. In a previously cited study, SARS-CoV vaccinated primates had greater pulmonary infiltration of an inflammatory phenotype macrophages (MAC387 +, CD163 + and HAM56-), with higher IL-6 production than unvaccinated ones. Interestingly, despite alternatively activated macrophages (M2) were reduced in number, they showed a 10-fold increase in IL-6 production, 8-fold in MCP-1 production and 5-fold in IL-8 production. This would ultimately enhance inflammation and monocytes/neutrophils chemotaxis [83].

Therefore, lung accumulation of IMM with atypical macrophage activation and higher pro-inflammatory cytokine expression could provide a feedback loop that culminates in CS development.

### Cytokine storm

CS is a serious life-threatening condition characterized by uncontrolled activation of macrophages and T cells, hypercytokinemia, hyperferritinemia that could finally cause multiple organ dysfunction. CS is the convergent pathway of several diseases that evolve with immune dysregulation and hyperinflammation [90]. The CS prototype is primary hemophagocytic lymphohistiocytosis (pHLH), caused by genetic defects that impair the cytotoxic capacity of TCD8^+^ lymphocytes and NK cells [53].

NK cells are necessary for immune regulation. Through recognition of upregulated stress ligands, they are able to eliminate activated macrophages and other myeloid cells. Impaired cytotoxic capacity leads to loss of regulation, with persistent cytokine production by myeloid cell. As previously mentioned, patients infected by SARS-CoV-2 had NK and TCD8^+^ cells with an exhausted phenotype, characterized by expression of the NKG2A receptor, which impairs its immunoregulatory mechanism [84].

Besides pHLH, several diseases, such as, cancer and infections can lead to CS, often termed secondary hemophagocytic lymphohistiocytosis (sHLH). When the underlying disease is autoimmune or autoinflammatory, sHLH is called macrophage activation syndrome (MAS) [53]. Viruses are the most common infectious trigger for sHLH, but they may also trigger pHLH. The most implicated viruses are DNA viruses, like Epstein-Baar virus and Cytomegalovirus, and less frequently RNA viruses, such as Dengue, Influenza, HIV and highly pathogenic coronavirus [53, 91–95]**.**

Regarding cytokine profile, COVID-19 severe cases have high IL-2R, IL-6, IL-10, TNF-α serum levels [51, 96–100], with conflicting data on IL-1β, IL-7, IL-8, IL-17, IFN-γ and G-CSF [101–105]. However, these findings must be cautiously interpreted since cytokine serum levels may not reflect tissue inflammatory process [106].

In a study comparing host response to SARS-CoV-2 with other viruses (i.e. MERS, SARS, Respiratory Syncytial Virus and Influenza A), a distinct transcriptional profile was shown in ex vivo human bronchial epithelium model. SARS-CoV-2 induced a low or absent type I and III interferon response, while it promoted a strong pro-inflammatory cytokine (IL-1β, IL-6) and chemokines MCP-1 and CXCL-8 expression, attracting monocytes and neutrophils, respectively [107]. Besides type I and III interferon, IFN-γ expression also tended to be lower in CD4 + T cells from severe cases compared to moderate ones [51].

**Fig. 3:**
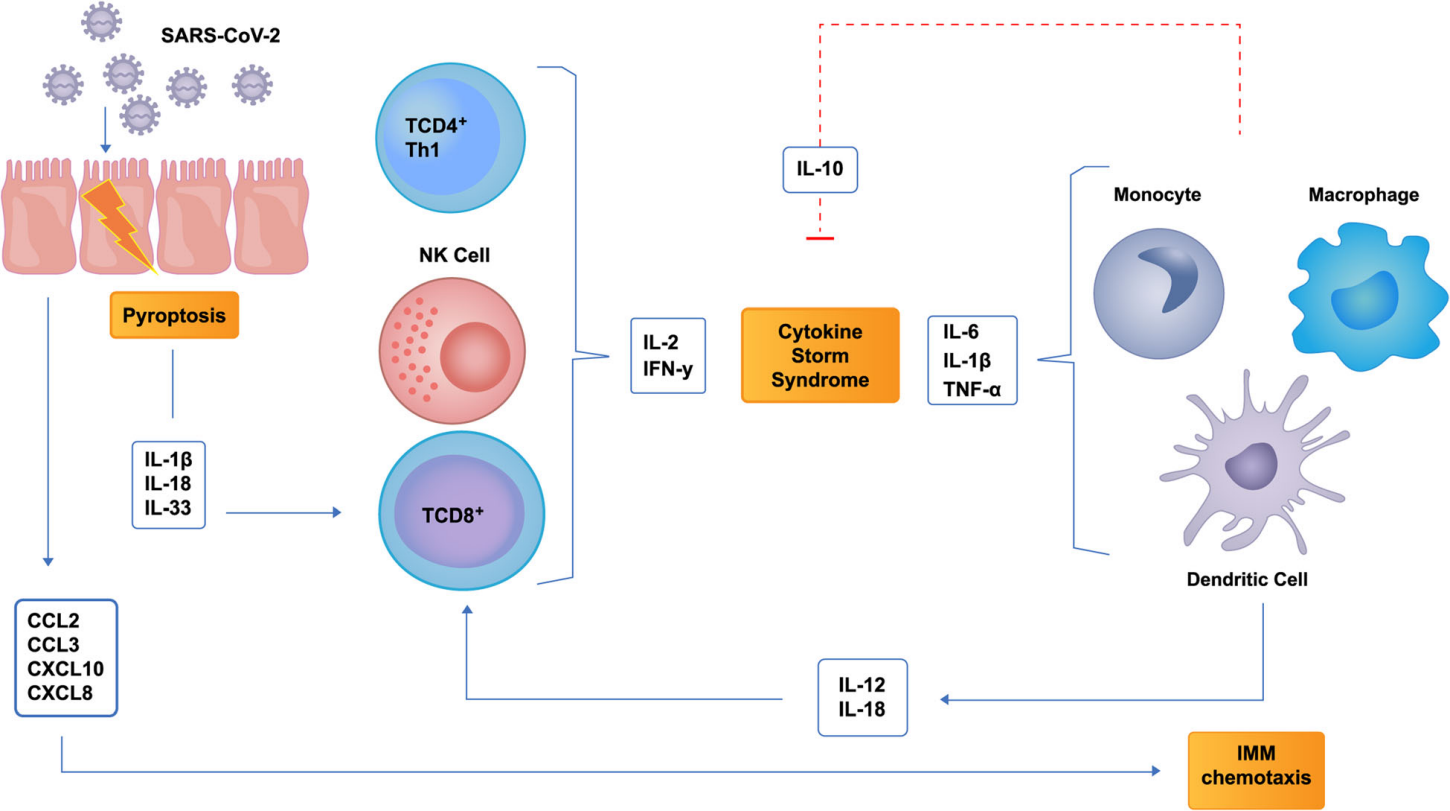
Cytokine Storm from SARS-CoV-2. SARS-CoV-2 infection cause pyroptosis of alveolar epithelial cells (type II pneumocytes) leading to release of cytokines such as IL-1 ß, IL-18 and IL-33 (alarmins). In turn, IL-1ß stimulates a large amount of pro-inflammatory cytokines and IL-18 stimulates IFN-γ release by TCD8^+^ effector cells, Th1 lymphocytes and NK Cells. Endothelial and epithelial damaged cells also release chemokines such as CCL2, CCL3 and CXCL10 that attract myeloid mononuclear cells that release more inflammatory cytokines, creating a positive feedback loop. IL-10 is released as an inefficient attempt to suppress inflammatory process

Data from SARS and MERS suggest that chemokines play a major role in inflammatory and immunity response to coronaviruses [93, 108]. Interferon-induced chemokines are crucial for an antiviral status. Among them, CXCL10 seems to be key for ARDS development as it has an important role on host viral defense [109, 110]. SARS-CoV infection of myeloid dendritic cells, even unproductive, leads to chemokines upregulation of CXCL10 (IP-10), CCL2 (MCP-1), CCL3 (MIP-1a) and CCL5 (RANTES) [111]. In a cluster of SARS-CoV-2 pneumonia, Huang et al. showed an upregulation of several chemokines in severe forms, such as CXCL10, CCL2 and CCL3 [96]. Thus, impaired interferon response seems to be related with a second wave of inflammation associated to lung monocyte infiltration, cytokine and chemokine production, and also tissue factor expression that leads to pro-thrombotic state [112] (Fig. 3).

Several studies are evaluating the effectiveness of treatments addressed to suppress CS, especially IL-6 inhibitors, such as tocilizumab and sarilumab. In some early case series, tocilizumab showed potential role to avoid mechanical ventilation and death [113, 114]. However, randomized clinical trials are important to demonstrate these beneficial effects. More recently, preliminary reports from randomized clinical trials failed to demonstrate a statistically significant benefit of IL-inhibitors. The COVACTA trial was interrupted due to side effects (opportunistic infections) [113, 115–117]. Probably this lack of benefit can be explained by the pleiotropic effect of pro-inflammatory cytokines.

The use of JAK inhibitors has been reported in clinical conditions with hypercytokininaemia, such as sHLH [118]. Because they act in the intracellular signaling pathway of several cytokines involved in CS, JAK inhibitors are potential treatments for severe forms of COVID-19. The main concern with its use is the inhibition of the antiviral response. Ongoing trials are evaluating the efficacy and safety of JAK inhibitors such as ruxolutinib and baricitinib in the treatment of COVID-19 [119, 120].

Up to date, dexamethasone is the only anti-inflammatory therapy that proves benefit in treatment of COVID-19 in a high quality randomized controlled trial. In preliminary report from RECOVERY trial, dexamethasone lower 28-day mortality among those who needs oxygen supplementation or respiratory support [121].

### Neutrophil extracellular traps

Neutrophil extracellular traps (NET) are web-like structures released by neutrophils composed by chromatin, histones, and granule proteins (e.g. neutrophil elastase, myeloperoxidase) that aim to trap pathogens or infected cells [122].

Prior studies linked excessive NET formation to tissue damage and pulmonary diseases, especially ARDS [123, 124]. NET can induce macrophage secretion of IL1β, further enhancing NET formation and CS [125, 126]. Viruses are known triggers of NET [127]. Considering its close relation to ARDS, and also that neutrophilia, high levels of IL-1β, IL-6, and D-dimer are poor outcome predictors in COVID-19, some authors suggest that NET may play a major role in its pathogenesis. Zuo et al. reported that patients with COVID-19 have high NET biomarkers serum levels, including cell-free DNA, myeloperoxidase DNA and citrullinated histone [122]. Moreover, NET can trigger microvascular thrombosis, leading to damage in the lungs, heart, and kidneys [122]. The interaction between NET and coagulation is addressed in details bellow.

### COVID-19 associated coagulopathy

The observation of high levels of fibrin degradation products in the serum of severe COVID-19 patients addressed the question on a specific coagulopathy in this context [8]. Lung necropsy of SARS patients had already showed diffuse alveolar damage along with small vessel thrombosis [128]. Similarly, pulmonary pathological analysis in COVID-19 revealed modest vessel wall immune cell infiltration with hyaline thrombosis and infarction [129].

Another study evaluated 19 patients with ARDS due to COVID-19, and evidenced a severe endothelial injury, the presence of intracellular virus in endothelial cells, with disrupted cell membranes and widespread thrombosis with microangiopathy [130]. Indeed, infection of endothelial cells expressing ACE2 by SARS-CoV-2 cause an endothelitis and with a massive release of plasminogen activator [14].

In COVID-19, fibrinogen is usually high as part of the acute phase response, but platelet count remains normal and severe coagulopathy is only seen in very severe and late-stage forms. Besides, high serum levels of D-dimer, mild or unchanged prothrombin time are also seen [131]. This differs from sepsis intravascular coagulation/ disseminated intravascular coagulation, since the latter usually evolves with thrombocytopenia and an increased prothrombin time [132].

Regardless of its clinical impact, coagulopathy seems to be triggered by hypercytokinemia [133]. Pro-inflammatory cytokines such as TNF-α and IL-6 induce tissue factor expression in monocytes and initiate coagulation cascade activation. In addition, these cytokines also suppress endogenous anticoagulant pathways [131]. MAS-like can cause local activation of endothelial cells from pulmonary vessels. The pro-inflammatory milieu leads to the upregulation of tissue factor and the reduction of fibrinolysis by Plasminogen activator inhibitor-1 [134].

In SARS-CoV associated coagulopathy, the high D-dimer levels seems to be result from upregulated urokinase-type plasminogen activator produced by alveolar macrophages [134].

Others proposed mechanisms for thrombotic phenomena in COVID-19 involves NET. First, it can activate the coagulation’s contact pathway and pulmonary megakaryocytes [8], through electrostatic interactions between histones and platelet phospholipids. Also, NET can process natural anticoagulant molecules, such as antithrombin III and tissue factor pathway inhibitor [103]. Thus, NET could link several aspects related to infection, inflammation and thrombosis in COVID-19 pathogenesis [135].

## Conclusions

Herein, we present the mechanisms of immune dysregulation, hyperinflammatory and immunothrombotic state reported to date in severe forms of COVID-19. Data from other coronaviruses regarding antibody-dependent enhancement may be a concern for vaccine development. Knowledge about kinetics of immune response and viral course are essential for patient care and could provide insights for immunomodulatory therapeutic strategies. Further studies correlating clinical and laboratorial data with immune status and viral load may help choosing properly suitable candidates for targeted immune therapy.

## Data Availability

All data generated or analyzed during this study are included in this published article [and its supplementary information files].

## Abbreviations

ACE2: Angiotensin-converting enzyme 2
ADE: Antibody-dependent enhancement
Ang-1: Angiotensin-1
Ang-2: Angiotensin-2
Ang-1-7: Angiotensin 1–7
Ang-1-9: Angiotensin 1–9
ARDS: Acute respiratory distress syndrome
DAMP: Damage associates molecular pattern
DHF: Dengue hemorrhagic fever
Nab: Neutralizing antibody
NET: Neutrophil extracellular traps
MAS: Macrophage Activation Syndrome
NLRP3: nucleotide-binding domain, leucine-rich repeat and pyrin domain-containing protein 3
PAMP: Pathogen associated molecular pattern
PRR: Pattern recognition receptors
pHLH: Primary hemophagocytic lymphohistiocytosis
sHLH: Secondary hemophagocytic lymphohistiocytosis

## References

[1] Baloch S, Baloch MA, Zheng T, Pei X (2020). The coronavirus disease 2019 (COVID-19) pandemic. Tohoku J Exp Med.

[2] Zhao Y, Zhao Z, Wang Y, Zhou Y, Ma Y, Zuo W. Single-cell RNA expression profiling of ACE2, the putative receptor of Wuhan 2019-nCov. bioRxiv. 2020:2020.01.26.919985 Available from: https://www.biorxiv.org/content/10.1101/2020.01.26.919985v1.

[3] Hoffmann M, Kleine-Weber H, Schroeder S, Krüger N, Herrler T, Erichsen S (2020). SARS-CoV-2 Cell Entry Depends on ACE2 and TMPRSS2 and Is Blocked by a Clinically Proven Protease Inhibitor. Cell.

[4] Romano M, Ruggiero A, Squeglia F, Maga G, Berisio R. A structural view of SARS-CoV-2 RNA replication machinery: RNA synthesis, proofreading and final capping. Cells. MDPI AG. 2020;9:1267. Available from: 10.3390/cells9051267. 10.3390/cells9051267 PMC7291026 32443810

[5] Tay MZ, Poh CM, Rénia L, MacAry PA, Ng LFP. The trinity of COVID-19: immunity, inflammation and intervention. Nat Rev Immunol. 2020:1–12 Available from: http://www.nature.com/articles/s41577-020-0311-8. 10.1038/s41577-020-0311-8 PMC7187672 32346093

[6] Wang D, Hu B, Hu C, et al. Clinical characteristics of 138 hospitalized patients with 2019 novel coronavirus-infected pneumonia in Wuhan, China. JAMA. 2020;323(11):1061–9. Available from: 10.1001/jama.2020.1585. 10.1001/jama.2020.1585 PMC7042881 32031570

[7] Ding, Q, Lu, P, Fan, Y, Xia, Y, Liu, M. The clinical characteristics of pneumonia patients coinfected with 2019 novel coronavirus and influenza virus in Wuhan, China. J Med Virol. 2020;92:1549–55. Avaible from: 10.1002/jmv.25781. 10.1002/jmv.25781 PMC7228290 32196707

[8] McGonagle D, O’Donnell J, Sharif K, Emery P, Bridgewood C (2020). Immune mechanisms of pulmonary intravascular coagulopathy (PIC) in COVID-19 pneumonia. Lancet Rheumatol.

[9] Xu H, Zhong L, Deng J, Peng J, Dan H, Zeng X (2020). High expression of ACE2 receptor of 2019-nCoV on the epithelial cells of oral mucosa. Int J Oral Sci.

[10] Devaux CA, Rolain JM, Colson P, Raoult D. New insights on the antiviral effects of chloroquine against coronavirus: what to expect for COVID-19? Int J Antimicrob Agents. 2020;55(5):105938. Available from: 10.1016/j.ijantimicag.2020.105938. 10.1016/j.ijantimicag.2020.105938 PMC7118659 32171740

[11] Geleris J, Sun Y, Platt J, et al. Observational study of hydroxychloroquine in hospitalized patients with Covid-19. N Engl J Med. 2020;382(25):2411–8. Avaible from: 10.1056/NEJMoa2012410. 10.1056/NEJMoa2012410 PMC7224609 32379955

[12] Boulware DR, Pullen MF, Bangdiwala AS, Pastick KA, Lofgren SM, Okafor EC (2020). A randomized trial of Hydroxychloroquine as Postexposure prophylaxis for Covid-19. N Engl J Med.

[13] Xu Z, Shi L, Wang Y, Zhang J, Huang L, Zhang C (2020). Pathological findings of COVID-19 associated with acute respiratory distress syndrome. Lancet Respir Med.

[14] Varga Z, Flammer AJ, Steiger P, Haberecker M, Andermatt R, Zinkernagel AS (2020). Endothelial cell infection and endotheliitis in COVID-19. Lancet.

[15] Man SM, Karki R, Kanneganti TD. Molecular mechanisms and functions of pyroptosis, inflammatory caspases and inflammasomes in infectious diseases. Immunol Rev. 2017;277(1):61–75. Available from: 10.1111/imr.12534. 10.1111/imr.12534 PMC5416822 28462526

[16] Yang M. Cell Pyroptosis, a potential pathogenic mechanism of 2019-nCoV infection. SSRN Electron J. 2020..

[17] Farag NS, Breitinger U, Breitinger HG, El Azizi MA. Viroporins and inflammasomes: a key to understand virus-induced inflammation. Int J Biochem Cell Biol. 2020;122:105738. Available from: 10.1016/j.biocel.2020.105738. 10.1016/j.biocel.2020.105738 PMC7102644 32156572

[18] Chen IY, Moriyama M, Chang MF, Ichinohe T (2019). Severe acute respiratory syndrome coronavirus viroporin 3a activates the NLRP3 inflammasome. Front Microbiol.

[19] Schoeman D, Fielding BC (2019). Coronavirus envelope protein: current knowledge. Virol J.

[20] Swanson KV, Deng M, Ting JPY (2019). The NLRP3 inflammasome: molecular activation and regulation to therapeutics. Nat Rev Immunol.

[21] El-Zayat SR, Sibaii H, Mannaa FA. Toll-like receptors activation, signaling, and targeting: an overview. Bull Natl Res Cent. 2019;43:187. Available from: 10.1186/s42269-019-0227-2.

[22] Limagne E, Lançon A, Delmas D, Cherkaoui-Malki M, Latruffe N (2016). Resveratrol interferes with IL1-β-induced pro-inflammatory paracrine interaction between primary chondrocytes and macrophages. Nutrients.

[23] Lythgoe MP, Middleton P (2020). Ongoing clinical trials for the management of the COVID-19 pandemic. Trends Pharmacol Sci.

[24] Huet T, Beaussier H, Voisin O, Jouveshomme S, Dauriat G, Lazareth I (2020). Anakinra for severe forms of COVID-19: a cohort study. Lancet Rheumatol.

[25] Schlesinger N, Firestein BL, Brunetti L (2020). Colchicine in COVID-19: an old drug. New Use.

[26] Patel VB, Zhong JC, Grant MB, Oudit GY (2016). Role of the ACE2/angiotensin 1-7 axis of the renin-angiotensin system in heart failure. Circ Res.

[27] Wang K, Gheblawi M, Oudit GY. Angiotensin converting enzyme 2: A double-edged sword. Circulation. 2020:1–8. 10.1161/CIRCULATIONAHA.120.047049 32213097

[28] Yan T, Xiao R, Lin G. Angiotensin-converting enzyme 2 in severe acute respiratory syndrome coronavirus and SARS-CoV-2: a double-edged sword? FASEB J. 2020;34(5):6017–26. Avaible from: 10.1096/fj.202000782. 10.1096/fj.202000782 PMC7264803 32306452

[29] Imai Y, Kuba K, Rao S, Huan Y, Guo F, Guan B (2005). Angiotensin-converting enzyme 2 protects from severe acute lung failure. Nature.

[30] Kuba K, Imai Y, Rao S, Gao H, Guo F, Guan B (2005). A crucial role of angiotensin converting enzyme 2 (ACE2) in SARS coronavirus-induced lung injury. Nat Med.

[31] Zhang Q, Cong M, Wang N, et al. Association of angiotensin-converting enzyme 2 gene polymorphism and enzymatic activity with essential hypertension in different gender: a case-control study. Medicine (Baltimore). 2018;97(42):e12917. Available from: 10.1097/MD.0000000000012917. 10.1097/MD.0000000000012917 PMC6211892 30335025

[32] da Silva JS, Gabriel-Costa D, Wang H, et al. Blunting of cardioprotective actions of estrogen in female rodent heart linked to altered expression of cardiac tissue chymase and ACE2. J Renin Angiotensin Aldosterone Syst. 2017;18(3):1470320317722270. Available from: 10.1177/1470320317722270. 10.1177/1470320317722270 PMC5805468 28748720

[33] Ciaglia E, Vecchione C, Puca AA (2020). COVID-19 infection and circulating ACE2 levels: protective role in women and children. Front Pediatr.

[34] Molony RD, Nguyen JT, Kong Y, Montgomery RR, Shaw AC, Iwasaki A (2017). Aging impairs both primary and secondary RIG-I signaling for interferon induction in human monocytes. Sci Signal.

[35] Annsea Park AI. Type I and Type III Interferons – Induction, Signaling, Evasion, and Application to Combat COVID-19. Cell Host Microbe. 2020:10;27(6):870–8. Available from: 10.1016/j.chom.2020.05.008. 10.1016/j.chom.2020.05.008 PMC7255347 32464097

[36] Languedoc DU (2010). N RDEC, Lili Z, La RUEDE, Romaine C. overview of the immune response. J Allergy Clin Immunol.

[37] Iwasaki A, Medzhitov R (2015). Control of adaptive immunity by the innate immune system. Nat Immunol.

[38] Nedvetzki S, Sowinski S, Eagle RA, Harris J, Vély F, Pende D (2007). Reciprocal regulation of human natural killer cells and macrophages associated with distinct immune synapses. Blood.

[39] Aiello A, Farzaneh F, Candore G, Caruso C, Davinelli S, Gambino CM (2019). Immunosenescence and its hallmarks: How to oppose aging strategically? A review of potential options for therapeutic intervention. Front Immunol.

[40] Lanna A, Henson SM, Escors D, Akbar AN (2014). The kinase p38 activated by the metabolic regulator AMPK and scaffold TAB1 drives the senescence of human T cells. Nat Immunol.

[41] Franceschi C, Campisi J (2014). Chronic inflammation (Inflammaging) and its potential contribution to age-associated diseases. J Gerontol Ser A Biol Sci Med Sci.

[42] Smits SL, de Lang A, van den Brand JMA, Leijten LM, van IJcken WF, et al. Exacerbated innate host response to SARS-CoV in aged non-human primates. PLOS Pathogens. 2010;6(2):e1000756. Available from: 10.1371/journal.ppat.1000756. 10.1371/journal.ppat.1000756 PMC2816697 20140198

[43] Fagone P, Ciurleo R, Lombardo SD, Iacobello C, Palermo CI, Shoenfeld Y (2020). Transcriptional landscape of SARS-CoV-2 infection dismantles pathogenic pathways activated by the virus, proposes unique sex-specific differences and predicts tailored therapeutic strategies. Autoimmun Rev.

[44] Williamson E, Walker AJ, Bhaskaran KJ, Bacon S, Bates C, Morton CE, et al. OpenSAFELY: factors associated with COVID-19-related hospital death in the linked electronic health records of 17 million adult NHS patients. medRxiv. 2020:2020.05.06.20092999 Available from: http://medrxiv.org/content/early/2020/05/07/2020.05.06.20092999.abstract.

[45] Richardson S, Hirsch JS, Narasimhan M, Crawford JM, McGinn T, Davidson KW (2020). Presenting characteristics, comorbidities, and outcomes among 5700 patients hospitalized with COVID-19 in the new York City area. JAMA.

[46] Chiappetta S, Sharma AM, Bottino V, Stier C. COVID-19 and the role of chronic inflammation in patients with obesity. Int J Obes. 2020;20:–2 Available from: 10.1038/s41366-020-0597-4. 10.1038/s41366-020-0597-4 PMC7224343 32409680

[47] Honce R, Karlsson EA, Wohlgemuth N, Estrada LD, Meliopoulos VA, Yao J (2020). Obesity-related microenvironment promotes emergence of virulent influenza virus strains. MBio.

[48] Tanase DM, Gosav EM, Radu S, Ouatu A, Rezus C, Ciocoiu M, et al. Arterial hypertension and interleukins: potential therapeutic target or future diagnostic marker? Int J Hypertens. 2019;2019. 10.1155/2019/3159283 PMC6521461 31186952

[49] Gupta R, Ghosh A, Kumar A, Misra A. Since January 2020 Elsevier has created a COVID-19 resource centre with free information in English and Mandarin on the novel coronavirus COVID- 19 . The COVID-19 resource centre is hosted on Elsevier Connect , the company ’ s public news and information. 2020;(January).

[50] Randeria SN, Thomson GJA, Nell TA, Roberts T, Pretorius E (2019). Inflammatory cytokines in type 2 diabetes mellitus as facilitators of hypercoagulation and abnormal clot formation. Cardiovasc Diabetol.

[51] Chen G, Wu D, Guo W, Cao Y, Huang D, Wang H (2020). Clinical and immunologic features in severe and moderate coronavirus disease 2019. J Clin Invest.

[52] Zhou F, Yu T, Du R, Fan G, Liu Y, Liu Z (2020). Clinical course and risk factors for mortality of adult inpatients with COVID-19 in Wuhan, China: a retrospective cohort study. Lancet.

[53] Schulert GS, Cron RQ. The genetics of macrophage activation syndrome. Genes Immun. 2020; Available from: 10.1038/s41435-020-0098-4. 10.1038/s41435-020-0098-4 32291394

[54] Henry BM, De Oliveira MHS, Benoit S, Plebani M, Lippi G. Hematologic, biochemical and immune biomarker abnormalities associated with severe illness and mortality in coronavirus disease 2019 (COVID-19): a meta-analysis. Clin Chem Lab Med. 2020;58(7):1021–8. Available from: 10.1515/cclm-2020-0369. 10.1515/cclm-2020-0369 32286245

[55] Herold T, Jurinovic V, Arnreich C, Hellmuth JC, von Bergwelt-Baildon M, Klein M, et al. Level of IL-6 predicts respiratory failure in hospitalized symptomatic COVID-19 patients. medRxiv. 2020:2020.04.01.20047381 Available from: http://medrxiv.org/content/early/2020/04/10/2020.04.01.20047381.abstract.

[56] Kermali M, Khalsa RK, Pillai K, Ismail Z, Harky A (2020). The role of biomarkers in diagnosis of COVID-19 – A systematic review. Life Sci.

[57] Tang N, Li D, Wang X, Sun Z (2020). Abnormal coagulation parameters are associated with poor prognosis in patients with novel coronavirus pneumonia. J Thromb Haemost.

[58] Wang F, Nie J, Wang H, Zhao Q, Xiong Y, Deng L (2020). Characteristics of peripheral lymphocyte subset alteration in COVID-19 pneumonia. J Infect Dis.

[59] Misra DP, Agarwal V, Gasparyan AY, Zimba O. Rheumatologists’ perspective on coronavirus disease 19 (COVID-19) and potential therapeutic targets. Clin Rheumatol. 2020;19 Available from: http://www.ncbi.nlm.nih.gov/pubmed/32277367. 10.1007/s10067-020-05073-9 PMC7145936 32277367

[60] Rahimmanesh, I.; Kouhpayeh, S.; Khanahmad H. The Conceptual Framework for SARS-CoV-2 Related Lymphopenia. Prepr - not peer-reviewed. 2020;(April):1–29. 10.4103/abr.abr_303_20 PMC8977610 35386537

[61] Yang X, Yu Y, Xu J, Shu H, Xia J, Liu H (2020). Clinical course and outcomes of critically ill patients with SARS-CoV-2 pneumonia in Wuhan, China: a single-centered, retrospective, observational study. Lancet Respir Med.

[62] Hadjadj J, Yatim N, Barnabei L, Corneau A, Breillat P, Carlier N, et al. Impaired type I interferon activity and exacerbated inflammatory responses in severe Covid-19 patients. Science. 2020;369(6504):718–24. Available from: 10.1126/science.abc6027. 10.1126/science.abc6027 PMC7402632 32661059

[63] Chow KT, Gale M (2015). SnapShot: Interferon Signaling. Cell.

[64] Mora-Arias T, Amezcua-Guerra LM. Type III Interferons (lambda Interferons) in rheumatic autoimmune diseases. Arch Immunol Ther Exp (Warsz) [Internet]. 2020;68(1):1–10. Available from: 10.1007/s00005-019-00564-3. 10.1007/s00005-019-00564-3 31915933

[65] Ivashkiv LB (2018). IFNγ: signalling, epigenetics and roles in immunity, metabolism, disease and cancer immunotherapy. Nat Rev Immunol.

[66] Gordon S, Plüddemann A (2018). Macrophage clearance of apoptotic cells: A critical assessment. Front Immunol.

[67] Jansen JM, Gerlach T, Elbahesh H, Rimmelzwaan GF, Saletti G (2019). Influenza virus-specific CD4+ and CD8+ T cell-mediated immunity induced by infection and vaccination. J Clin Virol.

[68] Chu H, Chan JF-W, Wang Y, Yuen TT-T, Chai Y, Hou Y, et al. Comparative replication and immune activation profiles of SARS-CoV-2 and SARS-CoV in human lungs: an ex vivo study with implications for the pathogenesis of COVID-19. Clin Infect Dis. 2020; Available from: 10.1093/cid/ciaa410. 10.1093/cid/ciaa410 PMC7184390 32270184

[69] Angelini MM, Neuman BW, Buchmeier MJ (2014). Untangling membrane rearrangement in the nidovirales. DNA Cell Biol.

[70] Channappanavar R, Fehr AR, Vijay R, Mack M, Zhao J, Meyerholz DK (2016). Dysregulated type I interferon and inflammatory monocyte-macrophage responses cause lethal pneumonia in SARS-CoV-infected mice. Cell Host Microbe.

[71] Channappanavar R, Fehr AR, Zheng J, Wohlford-Lenane C, Abrahante JE, Mack M (2019). IFN-I response timing relative to virus replication determines MERS coronavirus infection outcomes. J Clin Invest.

[72] Peiris JSM, Chu CM, Cheng VCC, Chan KS, Hung IFN, Poon LLM (2003). Clinical progression and viral load in a community outbreak of coronavirus-associated SARS pneumonia: A prospective study. Lancet.

[73] Huang AT, Garcia-Carreras B, Hitchings MDT, Yang B, Katzelnick L, Rattigan SM, et al. A systematic review of antibody mediated immunity to coronaviruses: antibody kinetics, correlates of protection, and association of antibody responses with severity of disease. medRxiv . 2020 ;2020.04.14.20065771. Available from: http://medrxiv.org/content/early/2020/04/17/2020.04.14.20065771.abstract. 10.1038/s41467-020-18450-4 PMC7499300 32943637

[74] Sol M (2003). Cancel Tirado and Kyoung-Jin Yoon. Antibody-dependent enhancement of virus infection and disease. Viral Immunol.

[75] Flipse J, Diosa-Toro MA, Hoornweg TE, Van De Pol DPI, Urcuqui-Inchima S, Smit JM (2016). Antibody-dependent enhancement of dengue virus infection in primary human macrophages; balancing higher fusion against antiviral responses. Sci Rep.

[76] Oliveira RAS, de Oliveira-Filho EF, Fernandes AIV, Brito CAA, Marques ETA, Tenório MC (2019). Previous dengue or zika virus exposure can drive to infection enhancement or neutralisation of other flaviviruses. Mem Inst Oswaldo Cruz.

[77] Kuzmina NA, Younan P, Gilchuk P, Santos RI, Flyak AI, Ilinykh PA (2018). Antibody-dependent enhancement of Ebola virus infection by human antibodies isolated from survivors. Cell Rep.

[78] Winarski KL, Tang J, Klenow L, Lee J, Coyle EM, Manischewitz J (2019). Antibody-dependent enhancement of influenza disease promoted by increase in hemagglutinin stem flexibility and virus fusion kinetics. Proc Natl Acad Sci U S A.

[79] Takano T, Nakaguchi M, Doki T, Hohdatsu T (2017). Antibody-dependent enhancement of serotype II feline enteric coronavirus infection in primary feline monocytes. Arch Virol.

[80] Dominguez SR, Robinson CC, Holmes KV (2009). Detection of four human coronaviruses in respiratory infections in children: A one-year study in Colorado. J Med Virol.

[81] Tseng C-TK, Perrone LA, Zhu H, Makino S, Peters CJ (2005). Severe acute respiratory syndrome and the innate immune responses: modulation of effector cell function without productive infection. J Immunol.

[82] Iwasaki A, Yang Y. The potential danger of suboptimal antibody responses in COVID-19. Nat Rev Immunol. 2020:1–3 Available from: 10.1038/s41577-020-0321-6. 10.1038/s41577-020-0321-6 PMC7187142 32317716

[83] Liu L, Wei Q, Lin Q, et al. Anti-spike IgG causes severe acute lung injury by skewing macrophage responses during acute SARS-CoV infection. JCI Insight. 2019;4(4):e123158. Available from: 10.1172/jci.insight.123158. 10.1172/jci.insight.123158 PMC6478436 30830861

[84] Zheng M, Gao Y, Wang G, Song G, Liu S, Sun D, et al. Functional exhaustion of antiviral lymphocytes in COVID-19 patients. Cell Mol Immunol. 2020;(March):7–9 Available from: 10.1038/s41423-020-0402-2. 10.1038/s41423-020-0402-2 PMC7091858 32203188

[85] Zheng HY, Zhang M, Yang CX, Zhang N, Wang XC, Yang XP (2020). Elevated exhaustion levels and reduced functional diversity of T cells in peripheral blood may predict severe progression in COVID-19 patients. Cell Mol Immunol.

[86] Doering TA, Crawford A, Angelosanto JM, Paley MA, Ziegler CG, Wherry EJ (2012). Network analysis reveals centrally connected genes and pathways involved in CD8+ T cell exhaustion versus memory. Immunity.

[87] Schönrich Günther, Raftery Martin J. The PD-1/PD-L1 axis and virus infections: a delicate balance. Front Cell Infect. Microbiol. 2019;9:207. Available from: 10.3389/fcimb.2019.00207. 10.3389/fcimb.2019.00207 PMC6584848 31263684

[88] Barber DL, Wherry EJ, Masopust D, Zhu B, Allison JP, Sharpe AH (2006). Restoring function in exhausted CD8 T cells during chronic viral infection. Nature.

[89] Xiaohong Y, Tingyuan L, Zhicheng H, Yifang P, Huawen L, Shicang Y (2020). A Histopathological study on the multi-site puncture of the new coronavirus pneumonia (COVID-19) in 3 cases. Chinese J Pathol.

[90] Brisse E, Wouters CH, Andrei G, Matthys P (2017). How viruses contribute to the pathogenesis of hemophagocytic lymphohistiocytosis. Front Immunol.

[91] Marsh RA (2018). Epstein-Barr virus and hemophagocytic lymphohistiocytosis. Front Immunol.

[92] Huang KJ, Su IJ, Theron M, Wu YC, Lai SK, Liu CC (2005). An interferon-γ-related cytokine storm in SARS patients. J Med Virol.

[93] Channappanavar R, Perlman S (2017). Pathogenic human coronavirus infections: causes and consequences of cytokine storm and immunopathology. Semin Immunopathol.

[94] Asanad S, Cerk B, Ramirez V (2018). Hemophagocytic lymphohistiocytosis (HLH) secondary to disseminated histoplasmosis in the setting of acquired immunodeficiency syndrome (AIDS). Med Mycol Case Rep.

[95] Beutel G, Wiesner O, Eder M, Hafer C, Schneider AS, Kielstein JT (2011). Virus-associated hemophagocytic syndrome as a major contributor to death in patients with 2009 influenza A (H1N1) infection. Crit Care.

[96] Huang C, Wang Y, Li X, Ren L, Zhao J, Hu Y (2020). Clinical features of patients infected with 2019 novel coronavirus in Wuhan, China. Lancet.

[97] Li X, Xu S, Yu M, Wang K, Tao Y, Zhou Y, et al. Risk factors for severity and mortality in adult COVID-19 inpatients in Wuhan. J Allergy Clin Immunol. 2020; Available from: 10.1016/j.jaci.2020.04.006. 10.1016/j.jaci.2020.04.006 PMC7152876 32294485

[98] Wang F, Hou H, Luo Y, Tang G, Wu S, Huang M, et al. The laboratory tests and host immunity of COVID-19 patients with different severity of illness. JCI Insight. 2020;5(10):e137799. Available from: 10.1172/jci.insight.137799. 10.1172/jci.insight.137799 PMC7259533 32324595

[99] Henderson LA, Canna SW, Schulert GS, Volpi S, Lee PY, Kernan KF, et al. On the alert for cytokine storm: immunopathology in COVID‐19. Arthritis Rheumatol. 2020;72:1059–63. Avaible from: 10.1002/art.41285. 10.1002/art.41285 PMC7262347 32293098

[100] Zhang B, Zhou X, Zhu C, Feng F, Qiu Y, Feng J, et al. Immune phenotyping based on neutrophil-to-lymphocyte ratio and IgG predicts disease severity and outcome for patients with COVID-19. medRxiv. 2020:2020.03.12.20035048 Available from: http://medrxiv.org/content/early/2020/03/16/2020.03.12.20035048.abstract. 10.3389/fmolb.2020.00157 PMC7350507 32719810

[101] Liu J, Li S, Liu J, Liang B, Wang X, Wang H, et al. Longitudinal characteristics of lymphocyte responses and cytokine profiles in the peripheral blood of SARS-CoV-2 infected patients. medRxiv. 2020; 2020.02.16.20023671. Available from: http://medrxiv.org/content/early/2020/02/22/2020.02.16.20023671.abstract. 10.1016/j.ebiom.2020.102763 PMC7165294 32361250

[102] Liu Y, Liao W, Wan L, Xiang T, Zhang W (2020). Correlation between relative nasopharyngeal virus RNA load and lymphocyte count disease severity in patients with COVID-19. Viral Immunol.

[103] Barnes BJ, Adrover JM, Baxter-Stoltzfus A, Borczuk A, Cools-Lartigue J, Crawford JM (2020). Targeting potential drivers of COVID-19: neutrophil extracellular traps. J Exp Med.

[104] Ouyang Y, Yin J, Wang W, Shi H, Shi Y, Xu B, et al. Down-regulated gene expression spectrum and immune responses changed during the disease progression in COVID-19 patients. Clin Infect Dis. 2020; Available from: 10.1093/cid/ciaa462. 10.1093/cid/ciaa462 PMC7188184 32307550

[105] Yang Y, Shen C, Li J, Yuan J, Yang M, Wang F, et al. Exuberant elevation of IP-10, MCP-3 and IL-1ra during SARS-CoV-2 infection is associated with disease severity and fatal outcome. medRxiv. 2020:2020.03.02.20029975 Available from: http://medrxiv.org/content/early/2020/03/06/2020.03.02.20029975.abstract.

[106] Jordan M, Prof FL, Allen C, De Benedetti F, Grom AA, Ballabio M (2015). A Novel Targeted Approach to the Treatment of Hemophagocytic Lymphohistiocytosis (HLH) with an Anti-Interferon Gamma (IFNγ) Monoclonal Antibody (mAb), NI-0501: First Results from a Pilot Phase 2 Study in Children with Primary HLH. Blood.

[107] Blanco-Melo D, Nilsson-Payant BE, Liu WC, Uhl S, Hoagland D, Møller R, et al. tenOever. Imbalanced host response to SARS-CoV-2 drives development of COVID-19. Cell. 2020;181(5)1036–45. Available from: 10.1016/j.cell.2020.04.026. 10.1016/j.cell.2020.04.026 PMC7227586 32416070

[108] Li G, Fan Y, Lai Y, Han T, Li Z, Zhou P (2020). Coronavirus infections and immune responses. J Med Virol.

[109] Hayney MS, Henriquez KM, Barnet JH, Ewers T, Champion HM, Flannery S (2017). Serum IFN-γ-induced protein 10 (IP-10) as a biomarker for severity of acute respiratory infection in healthy adults. J Clin Virol.

[110] Ichikawa A, Kuba K, Morita M, Chida S, Tezuka H, Hara H (2013). CXCL10-CXCR3 enhances the development of neutrophil-mediated fulminant lung injury of viral and nonviral origin. Am J Respir Crit Care Med.

[111] Law HK, Cheung CY, Ng HY, et al. Chemokine up-regulation in SARS-coronavirus-infected, monocyte-derived human dendritic cells. Blood. 2005;106(7):2366–74. Available from: 10.1182/blood-2004-10-4166. 10.1182/blood-2004-10-4166 PMC1895271 15860669

[112] McGonagle D, Sharif K, O'Regan A, Bridgewood C. The role of cytokines including Interleukin-6 in COVID-19 induced pneumonia and macrophage activation syndrome-like disease. 2020;19(6):102537. Available from: 10.1016/j.autrev.2020.102537. 10.1016/j.autrev.2020.102537 PMC7195002 32251717

[113] Luo P, Liu Y, Qiu L, Liu X, Liu D, Li J (2020). Tocilizumab treatment in COVID-19: A single center experience. J Med Virol.

[114] Michot J-M, Albiges L, Chaput N, Saada V, Pommeret F, Griscelli F, et al. Tocilizumab, an anti-IL6 receptor antibody, to treat Covid-19-related respiratory failure: a case report. Ann Oncol. 2020;(January):19–21 Available from: https://linkinghub.elsevier.com/retrieve/pii/S0923753420363870. 10.1016/j.annonc.2020.03.300 PMC7136869 32247642

[115] Xu X, Han M, Li T, Sun W, Wang D, Fu B (2020). Effective treatment of severe COVID-19 patients with tocilizumab. Proc Natl Acad Sci U S A.

[116] Toniati P, Piva S, Cattalini M, Garrafa E, Regola F, Castelli F (2020). Tocilizumab for the treatment of severe COVID-19 pneumonia with hyperinflammatory syndrome and acute respiratory failure: A single center study of 100 patients in Brescia, Italy. Autoimmun Rev.

[117] Radbel J, Narayanan N, Bhatt PJ (2020). Use of Tocilizumab for COVID-19-induced cytokine release syndrome: A cautionary case report. Chest.

[118] Ahmed A, Merrill SA, Alsawah F, Bockenstedt P, Campagnaro E, Devata S (2019). Ruxolitinib in adult patients with secondary haemophagocytic lymphohistiocytosis: an open-label, single-Centre, pilot trial. Lancet Haematol.

[119] La Rosée F, Bremer HC, Gehrke I, Kehr A, Hochhaus A, Birndt S (2020). The Janus kinase 1/2 inhibitor ruxolitinib in COVID-19 with severe systemic hyperinflammation. Leukemia.

[120] Cantini F, Niccoli L, Matarrese D, Nicastri E, Stobbione P, Goletti D (2020). Baricitinib therapy in COVID-19: A pilot study on safety and clinical impact. J Inf Secur.

[121] RECOVERY Collaborative Group, Horby P, Lim WS, et al. Dexamethasone in Hospitalized Patients with Covid-19 - Preliminary Report [published online ahead of print, 2020. N Engl J Med. 2020;NEJMoa2021436. 10.1056/NEJMoa2021436.

[122] Zuo Y, Yalavarthi S, Shi H, Gockman K, Zuo M, Madison JA, et al. Neutrophil extracellular traps in COVID-19. JCI Insight. 2020;5(11):e138999. Available from: 10.1172/jci.insight.138999. 10.1172/jci.insight.138999 PMC7308057 32329756

[123] Lee H, Pan P (2018). Neutrophil extracellular traps promoted alveolar macrophages pyroptosis in LPS induced ALI/ARDS. Eur Respir J.

[124] Twaddell SH, Baines KJ, Grainge C, Gibson PG (2019). The emerging role of neutrophil extracellular traps in respiratory disease. Chest.

[125] Hu Z, Murakami T, Tamura H, Reich J, Kuwahara-Arai K, Iba T (2017). Neutrophil extracellular traps induce IL-1β production by macrophages in combination with lipopolysaccharide. Int J Mol Med.

[126] Chen L, Zhao Y, Lai D, Zhang P, Yang Y, Li Y, et al. Neutrophil extracellular traps promote macrophage pyroptosis in sepsis article. Cell Death Dis. 2018;9(6) Available from: 10.1038/s41419-018-0538-5. 10.1038/s41419-018-0538-5 PMC5964241 29789550

[127] Shah RD, Wunderink RG (2017). Viral pneumonia and acute respiratory distress syndrome. Clin Chest Med.

[128] Ding Y, Wang H, Shen H, Li Z, Geng J, Han H (2003). The clinical pathology of severe acute respiratory syndrome (SARS): A report from China. J Pathol.

[129] Fox SE, Akmatbekov A, Harbert JL, Li G, Brown JQ, Vander Heide RS (2020). Pulmonary and Cardiac Pathology in Covid-19: The First Autopsy Series from New Orleans. medRxiv.

[130] Ackermann M, Verleden SE, Kuehnel M, Haverich A, Welte T, Laenger F, et al. Pulmonary Vascular Endothelialitis, Thrombosis, and Angiogenesis in Covid-19. N Engl J Med. 2020:NEJMoa2015432 Available from: http://www.nejm.org/doi/10.1056/NEJMoa2015432. 10.1056/NEJMoa2015432 PMC7412750 32437596

[131] Levi M, Thachil J, Iba T, Levy JH (2020). Coagulation abnormalities and thrombosis in patients with COVID-19. Lancet Haematol.

[132] Iba T, Levy JH, Warkentin TE, Thachil J, van der Poll T, Levi M (2019). Diagnosis and management of sepsis-induced coagulopathy and disseminated intravascular coagulation. J Thromb Haemost.

[133] Levi M, van der Poll T (2017). Coagulation and sepsis. Thromb Res.

[134] Hofstra JJH, Haitsma JJ, Juffermans NP, Levi M, Schultz MJ (2008). The role of bronchoalveolar hemostasis in the pathogenesis of acute lung injury. Semin Thromb Hemost.

[135] Becker RC. COVID-19 update: Covid-19-associated coagulopathy. J Thromb Thrombolysis. 2020;15:1–14. Available from: 10.1007/s11239-020-02134-3. 10.1007/s11239-020-02134-3 PMC7225095 32415579

